# Effects of health intervention program on cardiometabolic risk profiles from health evaluation center in Asian population: a longitudinal study and propensity analysis

**DOI:** 10.1186/s12955-015-0325-2

**Published:** 2015-08-20

**Authors:** Chuan-Chuan Liu, Chung-Lieh Hung, Shou-Chuan Shih, Hung-Ju Ko, Ray-E Chang

**Affiliations:** The Institute of Health Policy and Management, College of Public Health, National Taiwan University, Taipei, Taiwan; Health Evaluation Center, Mackay Memorial Hospital, Taipei, Taiwan; Division of Cardiology, Department of Internal Medicine, Mackay Memorial Hospital, Taipei, Taiwan; Division of Gastroenterology, Department of Internal Medicine, Mackay Memorial Hospital, Taipei, Taiwan

**Keywords:** Health promotion, Cardiometabolic profiles, Propensity matching, Primary prevention

## Abstract

**Background:**

Health intervention program (HIP) based on diet and lifestyle modifications had been shown to improve cardiovascular risks. The effects of such program on a variety of cardiometabolic outcome measures conducted in a strict analysis remained relatively unexplored.

**Materials and methods:**

A total of 2,660 participants (mean age: 43.3 ± 10 years, 63.6 % male) underwent annual health survey from our health evaluation department. We implemented health intervention program (HIP) in which diet and lifestyle modifications including smoking cessation and advised physical activities were introduced. We further studied the effects of HIP on several cardiometabolic outcome measures including Framingham, metabolic scores and renal function in terms of Egfr with a mean follow-up period of 38.5 months. Propensity score (PS) matching (HIP vs non-HIP group) was used to avoid effects of case selection bias.

**Results:**

Totally 1,004 (502 subjects for each group) left after PS matching protocol (both HIP and non-HIP group). The HIP group showed significant decline of waist circumference (−1.46 ± 0.61, *p* = 0.016), post-prandial glucose (−6.77 ± 2.06, *p* = 0.001), and total cholesterol level (−4.42 ± 2.15, *p* = 0.04), with borderline increase in eGFR (1.72 ± 0.94, *p* = 0.068) after an average of 1.91 ± 1.14 year follow up period. Exercise behavior significantly increased for those who received HIP when compared to the non-HIP group (44.6 vs 52.4 %, *p* = 0.014). PS matching and difference-in-difference (DID) analysis further confirmed the beneficial effects of ATP III reduction by HIP (−0.36 ± 0.06, *p* < 0.05).

**Conclusion:**

We demonstrated in our study that several cardiometabolic profiles can be substantially improved after health intervention introduction at the health evaluation center, supporting the beneficial evidence of such health intervention programs implementation based on primary prevention view points.

**Electronic supplementary material:**

The online version of this article (doi:10.1186/s12955-015-0325-2) contains supplementary material, which is available to authorized users.

## Introduction

The total estimated financial burden of cardiovascular disease (CVD) for 2009 had reached $475.3 billion in the united states, [1] with the averaged lifetime cost of ischemic stroke in the US estimated to be $140,048. The unawareness of early stage weight control or life style and dietary modification so far remained as the major causes of subsequent development of CVD. Thus far, Framingham risk score (FRS), a fairly robust scoring system with well-validated longitudinal epidemiological data, and had been used broadly as a “gold-standard” in projecting future cardiovascular risks [1]. On the other hand, metabolic syndrome, [2, 3], which takes a cluster of metabolic derangements tightly linked to cardiovascular disorders with central obesity as key clinical feature, had been demonstrated to identify subject to adverse cardiovascular outcomes in the past decade [3–6]. Compared to FRS, cardiovascular risk stratification based on the metabolic scoring is composed of more modifiable cardiometabolic risk factors and prone to lifestyle modification and health intervention.

Health intervention programs (HIP), which actually comprised of health promotion, counseling and associated education programs adopted by several earlier healthcare organizations based on the modification of health behaviors and dietary control, is of extraordinary clinical value in disease prevention and therapeutic intervention [7–10]. Therefore, hospitals should be implemented with cost-effective primary preventive practices both for disease prevention and health promotion. The health evaluation center, an emerging new medicare unit nearest to primary and preventive medicine, should theoretically play an pivotal role to develop or to screen and provide educative care delivery for selected subjects in need. However, there remains a gap between the ideal implementation of health promotion programs and the actual efficacy in a large volume unit from real world practice.

The aim of this study was to investigate whether the health promotion implementation may lead to effective and significant effects on cardiometabolic risk profiles based on hospital health screening programs in a tertiary medical center.

## Methods

### Study background

In addition to the routine and traditional offer of standard physical examination, clinical information or biochemical data collection, a health intervention program (HIP) was developed and was further funded as a new component of our routine health evaluation procedure at the health evaluation center from a tertiary medical center in Northern Taipei, Taiwan in 2002. The health intervention program (HIP) from our health examination center includes the following 3 components:

1) health education guidance preceding a health evaluation; 2) establishment of health management profiles; and 3) follow-up care and tracking. Follow-up tracking is managed by health management specialists using the Health Management Information System. This system not only records all health evaluation test results, physician diagnoses, physician recommendations and dates and times of the referral appointments, but also provides a list of abnormal results and the annual health evaluation comparison for patients who had at least two consecutive visits. This system can also facilitate specialists to track and provide follow-up care. After initially running of nearly 2 years to get the system, we started to conduct a health promotion protocol in our health evaluation center.

### Study design

In our current study cohort, we analyzed the effects of such HIP implemented from those individuals who underwent two consecutive health evaluations between January 2004–2009. A physician specializing in family or internal medicine provides patients with a systematic explanation of their health evaluation report on the day following examinations. The content of the report includes test results, physical examination results, specialist recommendations, a summary and explanation of the results, and appropriate health education. Follow-up visits are scheduled with prescriptions done immediately as well as the referral and registration procedures performed on the same day. For patients who require follow up visits, appointment was made by health management specialists. Subjects not eligible and precluded in our health evaluation center may include unstable clinical conditions including acute decompensated heart failure, acute coronary syndrome, acute cerebrovascular event or renal replacement therapy. We intended to invite all subjects to participate our HIP program. However, since not all subjects were willing to participate this program, we further categorize the baseline sample into two groups: those who received health promotion interventions (HIP group) and those who did not (non-HIP group). In brief, HIP programs were characterized by both nutrition-based health education on diet habit and lifestyle modification as the following items: 1) education provided with the essential information on modifiable, diet-related disorders including less salt intake, to minimize food/diet exposure enriched in cholesterol and sugars, and 2) further highlighted the concept of lifestyle modification including increasing exercise or physical activities, smoking cessation, optimization of alcohol use, regular daily life schedule planning or tasks management to avoid staying up late. Education was provided by individualized instruction from special trainee/specialist in our center. A detailed study protocol and components were listed in Additional file 1: Table S1.

### Study subjects recruitment and flowchart

**Fig. 1:**
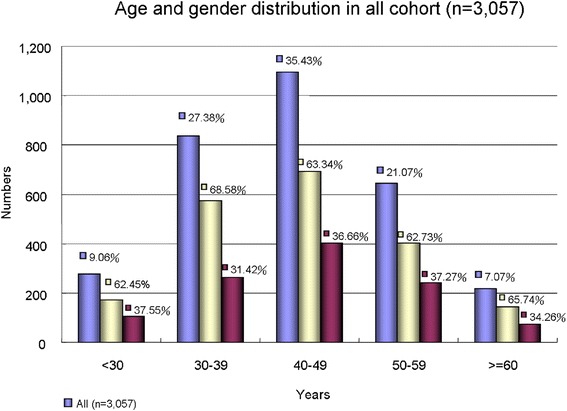
The age and gender distribution of original cohort from current study (n=3,057)

**Fig. 2:**
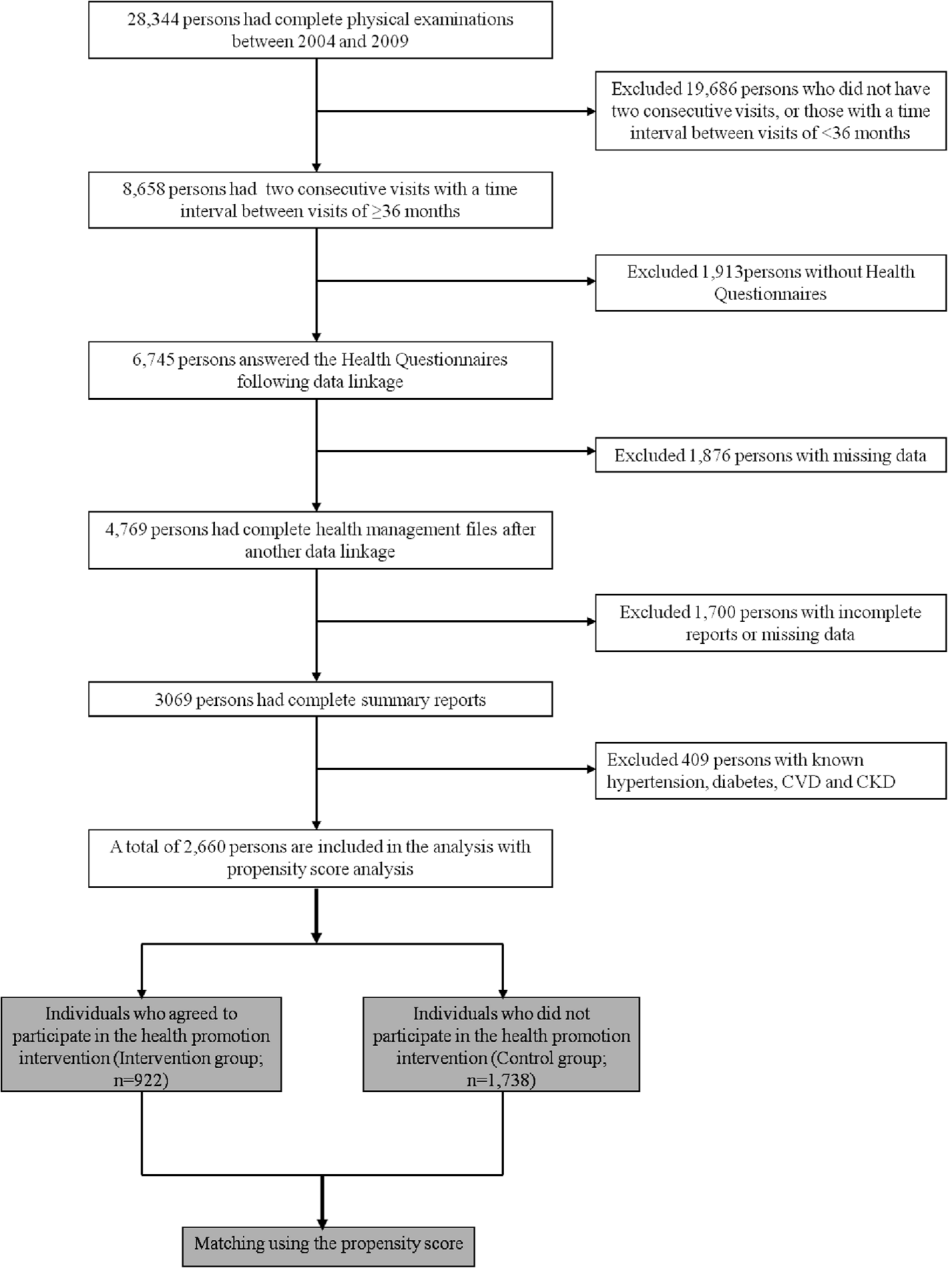
The flowchart of current study subjects and subjects excluded for final analysis and propensity matching

From the initial 28,344 persons who underwent health evaluation were eligible for our analysis, 19,686 persons who either had only one health evaluation or had a very short interval between two consecutive visits that was shorter than 36 months, leading to a total of 8,658 individuals remained. A total of 6,745 persons completed the questionnaires, while the remaining 1,913 persons were excluded. After further exclusion of those due to missing data (*n* = 1,876), incomplete reports (1,700), subjects with extreme values after descriptive data analysis (*n* = 12), the final cohort for this retrospective study consisted of 3,057 persons (Fig. 1). Among them, 922 subjects received HIP and 1,738 did not. The complete exclusion procedure of the sample is outlined in Fig. 2. The data collected from the first health evaluations constitutes the baseline, and the data collected from the second health evaluation constitutes the follow-up.

### Study procedure

The recipient of a health evaluation will track his or her health status according to the contents of the report and the physician’s recommendations, and will voluntarily follow-up with the department on an outpatient basis. Data considered includes physical examination questionnaires, satisfaction surveys, physiological and chemical examinations, metabolic scores (ATP III), FRS, eGFR, consultation notes from specialists, physical examination notes from physicians, records from case managers, follow-up care records, and the number of referrals and their associated costs. This study was approved by the local ethics committee of the Mackay Memorial hospital and patient information was anonymized and de-identified prior to analysis. This study received approval from the institutional review board (MMH-I-S-584) to perform retrospective research using secondary data.

### Outcome measures

1. The Assessment of Metabolic Scores: ATP III risk scores
  We assessed ATP III scores at baseline and at follow-up to quantify the change in health status among the intervention and control groups. A decrease in score indicates an improvement in health status, while an increase in score or no change in score indicates lack of improvement. Based on National Cholesterol Education Program-Adult Treatment Panel III (NCEP-ATP III) criteria score [11], we measured five risk indicators using a change score between 0 and 5, using components including: 1) abdominal obesity (waist circumference ≥90 cm and ≥80 cm in men and women, respectively); 2) low high-density lipoprotein cholesterol (HDL) (<40 mg/dl in men, <50 mg/dl in women); 3) high triglycerides (≥150 mg/dl); 4) high blood pressure (systolic blood pressure ≥130 mmHg or diastolic blood pressure ≥85 mmHg); 5) and high fasting glucose (≥100 mg/dl). According to the NCEP-ATP III score of 0–5, individuals with a score ≥3 are categorized as having the metabolic syndrome.
2. The Assessment of Framingham Risk Score (FRS)
  The operational definition of the cardiovascular risk assessment for Framingham Risk Score (FRS) included indicators of cardiovascular risk established in the National Cholesterol Education Program (NCEP) guidelines: age, total cholesterol, HDL cholesterol, blood pressure, and smoking status [11]. Based on the levels of risk factors, the percentage risk of developing chronic coronary heart disease (CHD) in the subsequent 5–10 years was quantified according to the conversion table. Subsequently, the cardiovascular risk can be delineated into high (>20 %), moderate (10–20 %), or low (<10 %) [11]. This study uses the percent change in cardiovascular risk score as the operational definition for health status improvement.
3. Estimated Glomerular Filtration Rate (eGFR)
  We relied on the Modification of Diet in Renal Disease (MDRD) in the definition of eGFR, which incorporated serum creatinine level into MDRD formula [12]. The percent change of eGFR from baseline to follow-up among the intervention and control groups was evaluated. An increase of eGFR score indicates health status deterioration, while a decrease indicates health status improvement.

### Statistical method

The analysis comparing the health status of the intervention and control groups was completed using SPSS 17.0 statistical software. Descriptive statistical analysis including the frequency distributions and percentages for all study variables were assessed for the entire sample and also according to group (intervention versus control). Inferential statistical analysis of propensity score analysis was performed by logistic regression. In order to avoid selection bias of several key factors in the comparison of intervention effects between HIP and non-HIP groups, we further matched the two groups based on a propensity score. The covariates included age, gender, smoking status, drinking status, exercise status, systolic blood pressure, diastolic blood pressure, BMI, ATP III score, FRS score, eGFR, blood glucose level, cholesterol, HDL cholesterol, LDL cholesterol, triglycerides, as well as family history of hypertension, diabetes, and cardiovascular diseases. We used the caliper and the radius matching methods. In order to prove the distribution of covariates among two groups is homogeneous, the matching distance was set at 0.05. This matching method reduces the effect of sampling bias and illustrates that the two groups consist of randomly assigned study samples. The matching algorithm for the propensity score (PS) was based on the caliper and radius matching methods, where the caliper signifies a tolerance level for the maximum distance in the propensity score. A distance of 0.05 was chosen for the current study. The use of PS ensures homogeneity in the baseline distributions of outcomes among the intervention and control groups, and also avoids estimation error caused by selection bias. Independent-samples *t*-test was used to compare the differences between the means of study continuous variables and risk indicators between groups with Chi-squared test used to evaluate proportional differences in categorical variables. Paired-*t* test and McNemar test were used to examine continuous and categorical variable changes about health improvement in the intervention or control group from baseline to follow-up.

Difference in difference (DID) analysis was introduced in our study, which first calculated the difference in a given outcome between baseline and follow-up for each of the intervention control groups to eliminate the potential biological changes over time. Subsequently, the changes in cardiovascular risk scores are compared to estimate the differences which may reflect the difference in the improvement of health status in one group (intervention group) over the other (non-intervention group).

## Results

### Baseline demographics

Of the initial 3,057 individuals participated the health screen program during study period (2004–2009; Additional file 1: Figure S1), 2,660 met eligible criteria and were selected for final enrollment (Additional file 1: Figure S1). Among them, 1,691 (63.6 %) were male and 969 (36.4 %) were female, resulting in a male to female ratio of 1.75:1. As shown in Table 1, the mean ± standard deviation (SD) age for the entire cohort was 43.3 ± 10.0 years, with resultant 922 and 1,738 subjects in the intervention and non-intervention groups, respectively (Additional file 1: Figure S2).

**Table 1:** Comparison of baseline data among study groups before and after propensity score matching

	Initial cohort before PSA matching (*N* = 2,660)			Cohort after PSA matching (*N* = 1004)		
Variable	Non-intervention (*n* = 1,738)	Intervention (*n* = 922)	*P* ^a^	Non-intervention (*n* = 502)	Intervention (*n* = 502)	*P* ^a^
Demographical data						
Age, year	40.93 (10.31)	44.85 (8.6)	<0.001	44.52 (9.35)	44.81 (8.14)	0.609
Male gender, %	65.4%	60.2%	0.008	62.9%	64.3%	0.646
Physiologic parameters						
Systolic blood pressure, mmHg	117.32(15.04)	116.68 (14.55)	0.293	117.03 (13.75)	117.57 (14.67)	0.549
Diastolic blood pressure, mmHg	73.43 (9.81)	73.45 (9.84)	0.946	73.34 (9.24)	73.86 (9.77)	0.387
Waist Circumference, cm	80.01 (10.18)	80.13 (9.59)	0.785	80.5 (9.3)	80.5 (9.8)	0.99
Body mass index, kg/m^2^	23.48 (3.32)	23.47 (3.15)	0.936	23.61 (2.86)	23.66 (3.21)	0.772
Serum sugar profiles						
Fasting glucose, mg/dL	92.96 (13.83)	94.73 (15.76)	0.0033	94.22 (16.95)	94.22 (13.54)	1.000
Post-prandial glucose, mg/dL	101.51 (27.92)	103.73 (30.92)	0.139	104.16 (33.06)	103.75 (28.91)	0.846
HbA1c, %	5.43 (0.61)	5.53 (0.58)	0.048	5.41 (0.53)	5.51 (0.58)	0.063
Lipid Profiles						
Total cholesterol, mg/dL	186.84 (32.90)	192.51 (33.42)	<0.001	191.16 (31.98)	192.06 (33.85)	0.666
HDL, mg/dL	54.07 (13.97)	56.10 (14.68)	0.006	55.42 (14.75)	55.76 (14.58)	0.713
LDL, mg/dL	118.46 (30.45)	124.86 (32.10)	<0.001	121.74 (29.55)	123.03 (31.11)	0.500
Triglyceride, mg/dL	110.05 (95.60)	115.96 (82.05)	0.151	112.83 (90.05)	113.45 (72.88)	0.917
Life style						
Smoking, %	11.0%	17.0%	<0.001	16.5%	17.3%	0.736
Alcohol Consumption, %	7.7%	18.3%	<0.001	16.1%	15.7%	0.863
Exercise, %	23.9%	48.2%	<0.001	43.6%	45.6%	0.526
Cardiometabolic Outcomes						
ATP III, number of MetS	1.28 (1.23)	1.49 (1.30)	<0.001	1.41 (1.27)	1.50 (1.27)	0.296
FRS, unit	4.57 (4.65)	4.97 (4.77)	0.132	4.68 (4.50)	4.76 (4.37)	0.809
eGFR, mL/min per 1.73 m^2^	86.05 (19.43)	84.30 (13.92)	0.028	83.51 (17.00)	83.88 (13.79)	0.704

^a^denotes either independent-sample t-test for continuous variable or chi-square test for categorical variable

When comparing the HIP to the non-HIP group on baseline demographics, lifestyle factors, medical histories, and cardiometabolic risk scoring before PS matching, those who underwent intervention tended to be older, less male gender, higher fasting glucose, HbA1c level, total cholesterol, HDL and LDL level, higher probability of smoking behavior, and alcohol use as well as regular exercise life styles (all *p* < 0.05). When compared the cardiometabolic risk scorings, the intervention group tended to have worse scorings when compared to the non-intervention group in terms of higher ATP III (1.49 vs 1.28) and lower eGFR, though the FRS did not show any significant differences between these two groups.

### Propensity Score (PS) analysis

A total of 502 subjects from each group were identified and following matching showed no significant differences in any of the covariates existed between the two groups. This indicates homogeneity between the intervention and control groups following PS matching. The results of the PS are presented in Table 1 (right columns), with the association between various baseline variables and propensity score demonstrated in Table 2. The C-statistic, as estimated by ROC curve, was 0.549. Model fitness was examined by the Hosmer and Lemeshow Goodness-of-Fit test, which indicates no significant differences between actual and model-predicted estimates (*p* = 0.311).

**Table 2:** The association between variables used to match with propensity score

Variable	Coefficient	Standard error	*P*
Age	0.003	0.008	0.649
Male gender	0.026	0.166	0.874
Systolic blood pressure, mmHg	0.002	0.005	0.743
Waist Circumference, cm	−0.01	0.006	0.115
Body mass index, kg/m2	0.007	0.025	0.790
Fasting glucose, mg/dL	−0.001	0.005	0.802
Total cholesterol, mg/dL	−0.016	0.010	0.100
HDL, mg/dL	0.016	0.009	0.077
LDL, mg/dL	0.017	0.010	0.086
Triglyceride, mg/dL	0.002	0.001	0.186
Smoking	0.069	0.185	0.710
Alcohol	−0.063	0.186	0.735
Exercise	0.049	0.132	0.710

Model fitness: C statistics = 0.549 *P* value of Hosmer and Lemeshow Goodness-of-Fit test = 0.311

In brief, there seemed no major differences regarding baseline demographic data such as age, gender distribution, blood pressures (both systolic and diastolic), body mass index, waist circumference, glucose level (fasting and post-prandial), HbA1c concentration, various lipid profiles (cholesterol, triglyceride, HDL and LDL), as well as life styles (smoking, exercise and alcohol consumption behaviors) (all p for differences > 0.05). In addition, we further demonstrated no differences of baseline cardiometabolic risk scorings including ATP III, FRS and eGFR (*p* = 0.296, 0.809 & 0.704, respectively).

### Baseline to follow-up differences in various clinical parameters and associated cardiometabolic risks

Results of baseline demographic and cardiometabolic risk profiles of the *t*-test assessing differences after propensity matching at follow-up between intervention and control groups are outlined in Table 3. For both intervention and non-intervention groups, there were no major differences regarding blood pressures (both systolic and diastolic), though significantly larger BMI (*p* = 0.01) and waist circumference were observed at the follow up visit in the non-intervention group (*p* = 0.012) with a significantly decrease in waist circumference in the intervention group (*p* < 0.001). Further, there were higher blood glucose level observed in the non-intervention group at the follow up visit, together with a higher HbA1c level (all *p* < 0.05). Furthermore, higher total cholesterol, LDL and triglyceride levels were observed at the follow up visit in the non-intervention group (both *p* < 0.05). Compared to the non-intervention group at follow up visit, we demonstrated a significantly reduction in waist circumference, post-prandial glucose, and total cholesterol level, resulting in significantly decrease of ATP III (−0.27 ± 0.08, *p* = 0.001) and a borderline improvement in eGFR.

**Table 3:** Difference between pre-test and post-test in each study group

	Non-intervention group (*n* = 502)			Intervention group (*n* = 502)			Difference in posttest data		
Variable	Pretest	Posttest	*P* ^a^	Pretest	Posttest	*P* ^a^	Mean	*SE*	*P* ^b^
Physiologic parameters									
Systolic blood pressure, mmHg	117.03 (13.75)	117.54 (14.85)	0.426	117.57 (14.67)	116.78 (13.94)	0.191	−0.76	0.91	0.403
Diastolic blood pressure, mmHg	73.34 (9.24)	73.48 (9.80)	0.719	73.86 (9.77)	73.88 (9.41)	1.000	0.40	0.61	0.513
Waist circumference, cm	80.5 (9.3)	80.98 (9.06)	0.012	80.5 (9.8)	79.51 (9.69)	<0.001	−1.46	0.61	0.016
Body mass index, kg/m^2^	23.61 (2.86)	23.72 (2.93)	0.010	23.66 (3.21)	23.66 (3.23)	0.922	−0.06	0.20	0.740
Serum sugar profiles									
Fasting glucose, mg/dL	94.22 (16.95)	95.95 (17.65)	0.001	94.22 (13.54)	95.12 (14.43)	0.079	−0.83	1.02	0.419
Post-prandial glucose, mg/dL	104.16 (33.06)	108.00 (33.89)	0.033	103.75 (28.91)	101.05 (25.45)	0.069	−6.77	2.06	0.001
HbA1c, %	5.41 (0.53)	5.69 (0.63)	<0.001	5.51 (0.58)	5.72 (0.70)	<0.001	0.04	0.06	0.524
Lipid Profiles									
Total cholesterol, mg/dL	191.16 (31.98)	195.30 (34.93)	<0.001	192.06 (33.85)	190.88 (32.78)	0.279	−4.42	2.15	0.040
HDL, mg/dL	55.42 (14.75)	56.10 (15.49)	0.110	55.76 (14.58)	55.21 (14.52)	0.039	−0.90	0.98	0.362
LDL, mg/dL	121.74 (29.55)	126.17 (32.39)	<0.001	123.03 (31.11)	124.35 (30.86)	0.131	−1.81	2.07	0.380
Triglyceride, mg/dL	112.83 (112.05)	120.07 (90.42)	0.009	113.45 (72.88)	119.16 (73.29)	0.021	−0.91	5.24	0.862
Cardiometabolic Outcomes									
ATP III, number of MetS	1.41 (1.27)	1.64 (1.29)	<0.001	1.50 (1.27)	1.37 (1.26)	0.005	−0.27	0.08	0.001
FRS, unit	4.68 (4.50)	5.36 (4.71)	0.005	4.76 (4.37)	5.24 (4.58)	0.025	−0.08	0.34	0.807
eGFR, mL/min per 1.73 m^2^	83.51 (17.00)	84.62 (14.95)	0.087	83.88 (13.79)	86.39 (14.64)	<0.001	1.72	0.94	0.068

Data not specified were presented as Mean (*SD*); ^a^denotes paired-sample t-test; ^b^denotes independent-sample t-test

### Difference-in-difference analysis of various clinical parameters and associated cardiometabolic risks at follow up

As presented in Table 4, the result of the DID analysis for various variables before and after intervention observation period showed that there was substantial reduction in waist circumference, post-prandial glucose, total cholesterol, higher HDL (all *p* < 0.05) and borderline reduction of BMI and LDL (*p* = 0.071 & 0.082, respectively) when comparing the intervention to the non-intervention group. As for life styles modification, we also demonstrated in the intervention group that exercise behavior significantly increased at the follow up visit (*p* = 0.007 within group), and showed significant differences when compared to the non-intervention group (*p* = 0.014 between groups). These changes of baseline variables between two groups over the observed study period in turn further resulted in substantially decrease of ATP III (−0.36 ± 0.06, *p* < 0.001) and a borderline increase in eGFR (1.39 ± 0.8, *p* = 0.084) when the intervention group was compared to the non-intervention population.

**Table 4:** Difference in difference models with results displayed comparing Non-intervention and Intervention Groups

	Non-intervention group (*n* = 502)		Intervention group (*n* = 502)		Difference in difference (Intervention minus non-intervention)		
Variable	Mean	*SD*	Mean	*SD*	Mean	*SE*	*P* ^a^
Physiologic parameters							
Systolic blood pressure, mmHg	0.53	14.92	−0.81	13.77	−1.34	0.91	0.141
Diastolic blood pressure, mmHg	0.16	10.08	0.00	9.73	−0.16	0.63	0.795
Waist Circumference, cm	0.72	5.95	−0.96	6.04	−1.69	0.4	<0.001
Body mass index, kg/m^2^	0.12	1.01	0.00	0.97	−0.11	0.06	0.071
Serum sugar profiles							
Fasting glucose, mg/dL	1.72	11.06	0.92	11.70	−0.79	0.72	0.271
Post-prandial glucose, mg/dL	3.74	32.28	−2.30	27.01	−6.04	2.10	0.004
HbA1c, %	0.26	0.33	0.22	0.62	−0.05	0.05	0.315
Lipid Profiles							
Total cholesterol, mg/dL	4.13	23.92	−1.08	22.16	−5.21	1.47	<0.001
HDL, mg/dL	−0.76	7.89	0.63	8.51	1.38	0.54	0.010
LDL, mg/dL	3.85	22.95	1.40	19.96	−2.45	1.41	0.082
Triglyceride, mg/dL	7.17	60.98	5.43	52.17	−1.74	3.61	0.630
Cardiometabolic Outcomes							
ATP III, number of MetS	0.23	1.02	−0.12	0.96	−0.36	0.06	<0.001
FRS, unit	0.55	3.49	0.41	3.24	−0.15	0.27	0.582
eGFR, mL/min per 1.73 m^2^	1.10	14.24	2.48	10.71	1.39	0.80	0.084

*SD* = standard deviation; *SE* = standard error of the mean difference; ^a^denotes independent-sample t-test

## Discussion

In the current study, we demonstrated that a strategic health promotion intervention based on HIP improved cardiovascular risks substantially in multiple dimensions, which included waist reduction, decrease in post-prandial glucose, cholesterol level as well as ATP III score, which still holds true after DID analysis (HIP versus non-HIP). The intervention programs also successfully enhanced the level of physical activity, when combined with the positive effects of previous biochemical and anthropometric data, further resulted in substantial reduction of metabolic scoring, and a borderline improvement of renal function in terms of eGFR. These results indicate that our health improvement intervention provides evidence-based cardiometabolic risks improvement in an Asian population during the 3 years HIP in this study.

Owing to the rapid growth of several common risk factors responsible for large invisible epidemic chronic diseases burden including unhealthy diet, physical inactivity and tobacco use, the World Health Organization (WHO) had undertaken a series of programs and set a goal to reduce the morbidity and mortality from such diseases [13–15]. So far, it is well-known that both health promotion and health education programs from previous studies had successfully demonstrated the effectiveness on disease prevention or the cease of disease progression, with special focus on indices related to metabolic scorings which had been shown to be tightly linked to community health [16, 17]. Tovar et al. had recently demonstrated that active control on diet contents may show beneficial effects on several cardiometabolic parameters including sugar level, several lipid profiles as well as blood pressure, which indicated the potentiality of dietary control based on the view points of primary preventive medicine [18]. Jahangiry et al. also ever published data regarding effects of lifestyle modification on metabolic syndrome in a dedicated designed randomized control trial, though in which study the case numbers are relatively small. In our current work, we similarly demonstrated that dietary modifications by active intervention showed positive effects on several lipid profiles, including drastic decline of total cholesterol, significant increase of HDL and borderline drop in LDL level, as well as improvement in post-prandial sugar control.

On the other hand, the significance of physical activity during lifestyle intervention had also been addressed and reported by Kujala et al., who observed a substantial decrease in both body weight and waist circumference for those at high risk for type 2 diabetes subjects but capable of carrying out higher physical activity level in the past 1 year [19]. The significant increase of exercise behavior and the substantial decrease of waist circumference and borderline decline of BMI at follow up visit in the HIP compared to the non-HIP group in our current study. In a previous report by Grundy et al., they showed that physical activity and weight reduction can act as first line therapy for metabolic syndrome [20, 21]. In our current work, we showed similar results in that HIP may effectively reduce metabolic scoring via increased physical activity and significantly reduced waist circumference, which was believed to be the key pathological component of metabolic syndrome. Our current data further extended beyond their findings in that we showed that these effects may be attributable to decreased total cholesterol, lowered post-prandial glucose as well as elevated HDL level.

Persistent physical training had been shown to be beneficial for prevention and disease management of diabetes and cardiovascular diseases [22–25]. Healthy diet controls, on the other hand, had also been shown to result in desirable health management results [26]. Of note, we showed that the clinical variables most subjective to the intervention therapy were waist circumference, body mass, post-prandial glucose level, renal function as well as several lipid profiles. There effects, when taken together, in part may help to explain the substantial improvement of metabolic scores at follow-up visit.

The strength of our work is the less biased nature of subjects enrolled for comparison between the HIP and non-HIP groups by utilizing propensity matching simulating randomized design from a relatively large Asian cohort since we fully acknowledged that potential selection bias in our work could not be avoided. The conduction of propensity matching may theoretically reduce the risks of baseline demographic distribution and the biochemical differences as much as possible. Furthermore, we conducted a propensity matching (PS) and difference-in-difference analysis (DID) which allows analysis of these effects less biased and to minimize the potential differences for those subjects with and without intervention during some period of follow-up. Even though, we still consistently showed the advantage of participating intervention programs in cardiometabolic risk reduction. To our best knowledge, there is a paucity of published studies that address relevant issues with relative large number of subjects enrolled from health evaluation center so far with meaningful results reported. While previous data on observational community-based reports seldom compare the potential effects of health promotion programs based on more strict study design with control group, our current data may provide invaluable information about the feasibility and usefulness of these programs implemented on a daily basis in tertiary care system [18, 27–30].

## Additional file

Additional file 1: Table S1. The major components, content of examinations, measurements and intervention programs in Health Intervention Program (HIP) in our center. Figure S1. As shown in Figure S1, the age and sex distribution of all 3,057 individuals in this health screen program before the final enrollment criteria. Figure S2. Among 2,660 individuals who fit the enrollment criteria before propensity matching, 1,691 (63.6 %) were male and 969 (36.4 %) were female, resulting in a total of 922 and 1,738 subjects in the intervention and non-intervention groups, respectively. (DOC 232 kb)
