# Supplementary material for: Effects of health intervention program on cardiometabolic risk profiles from health evaluation center in Asian population: a longitudinal study and propensity analysis
Source: Health Qual Life Outcomes. 2015 Aug 20;13:132. doi: 10.1186/s12955-015-0325-2 (PMC4545916; doi:10.1186/s12955-015-0325-2)

**Additional file 1**

**Table 1 The major components, content of examinations, measurements and intervention programs in Health Intervention Program (HIP) in our center**

| Content of Health Intervention Program (HIP) of Our Center | | | | | | | |
| --- | --- | --- | --- | --- | --- | --- | --- |
| Major Components | Health Education | Health Management Profiles | Follow-up Care |  |  | |  |
| Measurements or Data Collection |  |  |  |  |  | |  |
| Baseline Demographics | Age | Sex | Height | Weight | BMI | | Waist |
| Physical Examination | SBP | DBP | HR | Signs of Acute Clinical Conditions | | | |
| Biochemical Data |  |  |  |  |  |  | |
|  | Primary Measures |  |  |  |  |  | |
|  | Fasting glucose | Post-prandial glucose | Total cholesterol | Triglyceride | HDL | Creatinine | |
|  |  |  |  |  |  |  | |
|  | Secondary Measures |  |  |  |  |  | |
|  | HbA1c | LDL |  |  |  |  | |
| Lifestyles |  |  |  |  |  |  | |
|  | Smoking | Alcohol Consumption | Exercise |  |  |  | |
| Intervention Programs | Diet Control | Lifestyle Modification |  |  |  |  | |
| Study Period | January 2004 - 2009 |  |  |  |  |  | |
| Mean Time of Follow-up | 38.5 months |  |  |  |  |  | |
| Outcome Measures | ATP III | FRS | eGFR |  |  |  | |

ATP III: National Cholesterol Education Program-Adult Treatment Panel III, BMI: body mass index, DBP: diastolic blood pressure, eGFR: estimated glomerular filtration rate, FRS: Framingham Risk Score, HDL: high-density lipoprotein cholesterol, HR: heart rate, LDL: low-density lipoprotein cholesterol, MetS: metabolic SBP: systolic blood pressure.

Figure 1


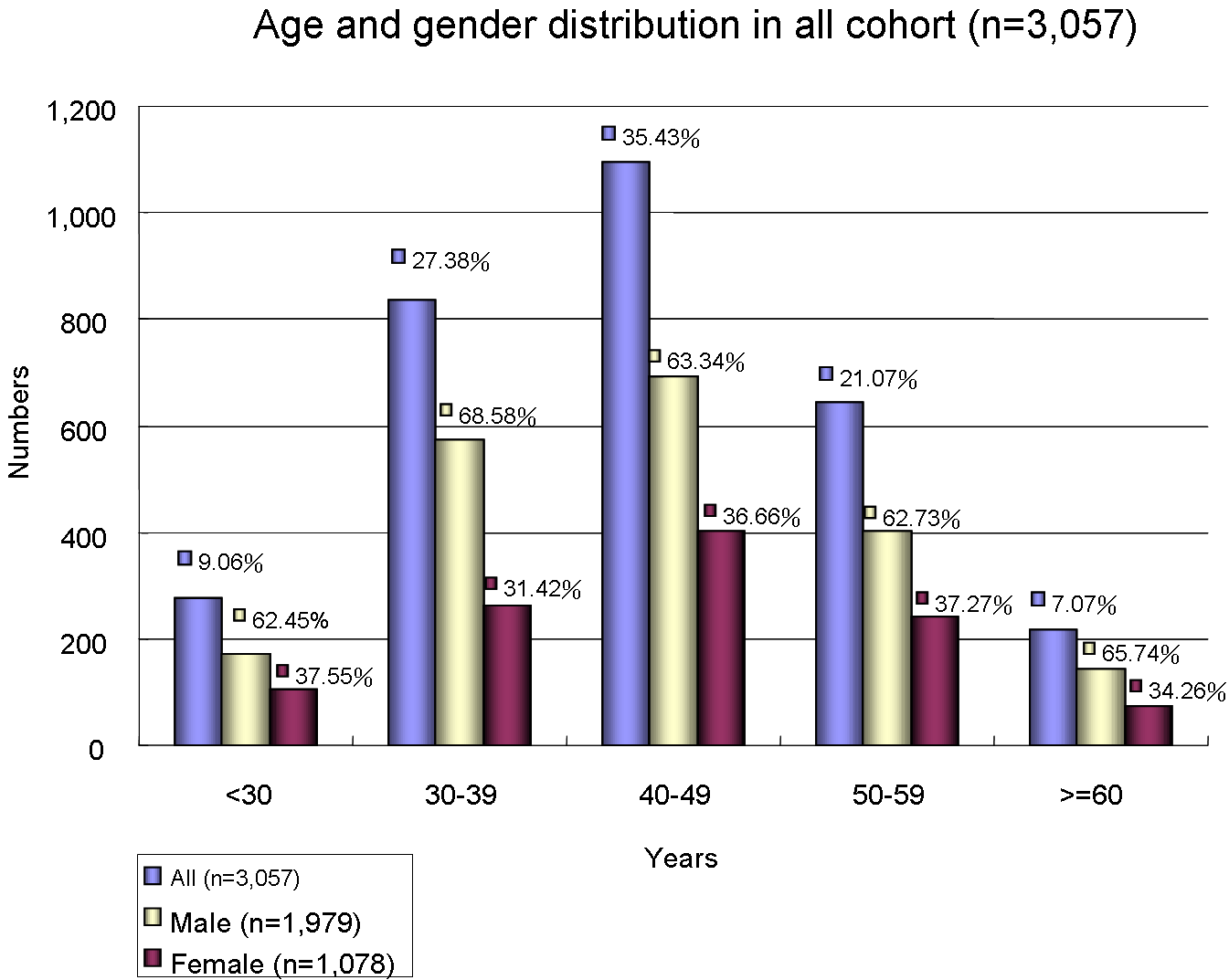


Figure 2


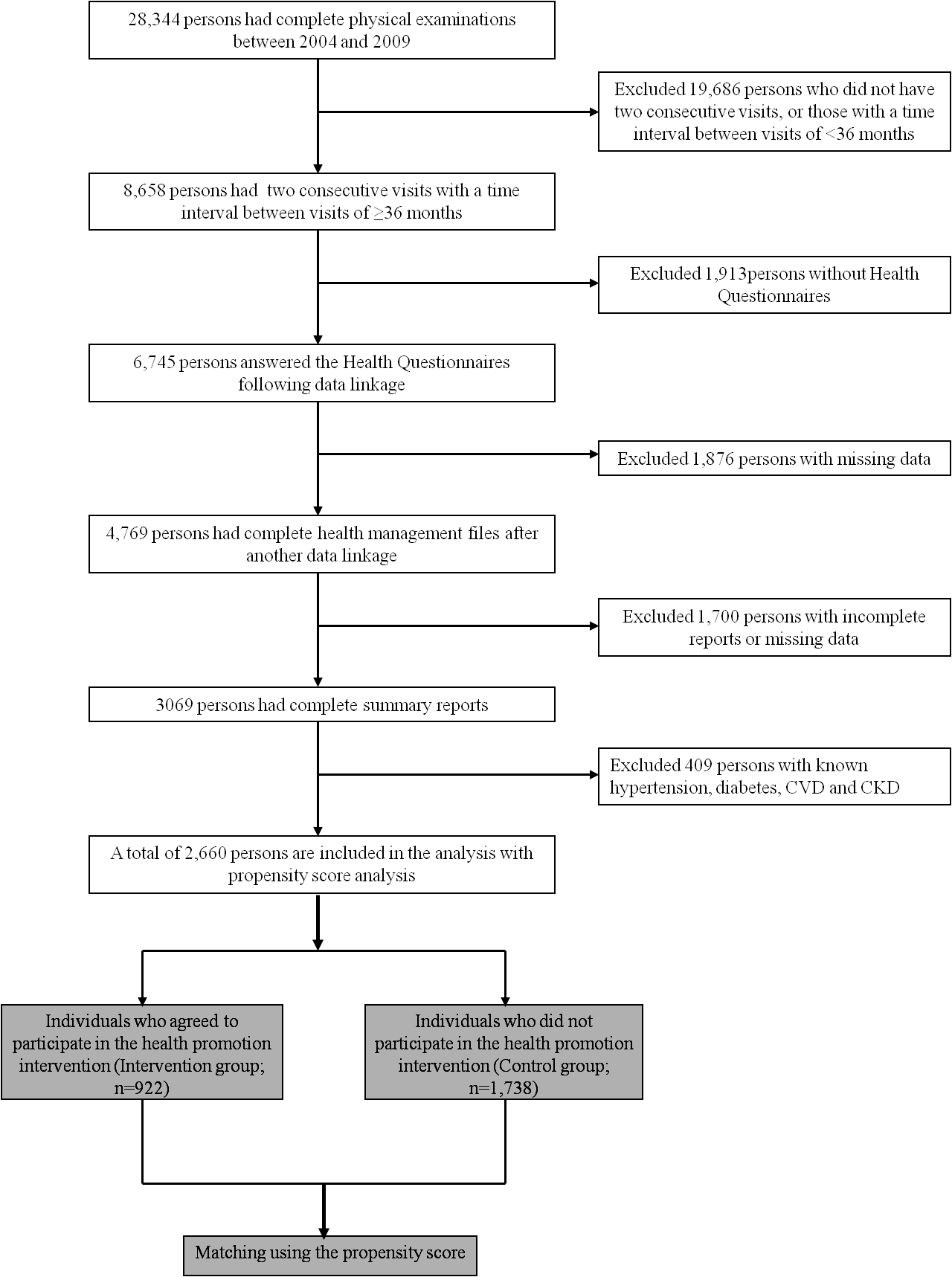

Supplement: Additional file 1: Table S1. — The major components, content of examinations, measurements and intervention programs in Health Intervention Program (HIP) in our center. Figure S1. As shown in Figure S1, the age and sex distribution of all 3,057 individuals in this health screen program before the final enrollment criteria. Figure S2. Among 2,660 individuals who fit the enrollment criteria before propensity matching, 1,691 (63.6 %) were male and 969 (36.4 %) were female, resulting in a total of 922 and 1,738 subjects in the intervention and non-intervention groups, respectively. (DOC 232 kb) [file 12955_2015_325_MOESM1_ESM.doc]
